# Long-Term Microplastic Accumulation Is Associated with Element-Specific Heavy Metal Mobilization in Agricultural Soils: Field Evidence from the Anning Valley, China

**DOI:** 10.3390/toxics14100863

**Published:** 2026-09-28

**Authors:** Yuqing Zhang, Guangli Xiao, Junlun Meng, Siwei Deng, Wenhao Wang, Xingguo Hua, Xuemei Gou, Tianwei He, Yi Wu

**Affiliations:** 1 Sichuan Academy of Eco-Environmental Sciences, Sichuan Province Engineering Technology Research Center of Emerging Contaminants Treatment and Environmental Health, Chengdu 610041, China; 2 College of Ecology and Environment, Chengdu University of Technology, Chengdu 610051, China; 3 State Key Laboratory of Geohazard Prevention and Geoenvironment Protection, Chengdu University of Technology, Chengdu 610059, China; 4 Sichuan Institute of Energetical and Geological Survey, Chengdu 610072, China

**Keywords:** soil microplastics, heavy metals, chemical fractionation, synergistic contamination, spatial heterogeneity, element-dependency

## Abstract

Microplastics (MPs) are increasingly recognized as emerging contaminants, making a systematic understanding of the co-occurrence patterns between MPs and conventional heavy metals (HMs) essential for addressing composite environmental risks. This study investigated MP abundance, morphology, and polymer composition, together with HM concentrations and chemical speciation in greenhouse farmlands across the Anning Valley, China, spanning different cultivation histories (1–8 years) and soil depths (0–40 cm). The results revealed that longer cultivation histories were accompanied by a marked accumulation of MPs, characterized by pronounced topsoil enrichment. MPs abundance increased from 758.53 n/kg in newly cultivated plots to 3373.19 n/kg in long-term plots, with topsoil level being 2.6 times higher than those in deeper layers (2462.25 vs. 951.25 n/kg). Under long-term cultivation, heavy metal contamination escalated, and contamination hotspots shifted from deeper soil to the MP-rich topsoil. In topsoil, HM also exhibited a higher proportion of potentially mobile fractions, reflecting greater potential mobility and bioavailability. Statistical associations between MPs and HMs co-varied with soil pH, organic matter, and coarse soil fractions, indicating complex inter-relationships influenced by shared agricultural management practices. This association weakened with soil depth and varied among elements, with Cd showing the strongest relationship with MPs and becoming the dominant contributor to HM risks. These findings demonstrate that farmland MP–HM co-contamination is spatially heterogeneous and metal-specific, providing field-scale evidence for improving ecological risk assessment and soil quality management under long-term plastic use.

## 1. Introduction

Microplastics (MPs, particles < 5 mm) could enter soil through agricultural practices, municipal waste, wastewater, atmospheric deposition, and construction waste [1], leading to reported soil MP abundances exceeding those in marine environments by more than 20-fold [2]. Soil MPs are persistent, resisting degradation and mineralization, and this leaves them in varying stages of incomplete decomposition with high morphological heterogeneity which further complicates their behavior in ecosystems [3]. Furthermore, laboratory studies show that MPs can adsorb co-existing contaminants (e.g., heavy metals) via electrostatic interactions, complexation, and cation bridging [4]. This interaction is reported to influence the environmental behavior, mobility, and bioavailability of other co-existing pollutants, potentially modulating their ecotoxicity and posing a threat to soil organisms and the food chain [5]. Therefore, systematically deciphering the interactions between MPs and other hazardous substances in soil is crucial for a comprehensive assessment of their integrated environmental risk.

Farmland has become a major terrestrial sink, with agricultural plastic use alone accounting for approximately 8 million tons annually [6]. Under long-term UV radiation, mechanical abrasion, and farming operations, materials such as mulch/greenhouse canopy films, irrigation pipes, ropes, seedling containers, and packaging materials continuously generate and release MPs, which could directly impact overall soil functioning [7]. Unlike freshly produced plastics, environmental MPs continuously undergo physical and chemical aging, resulting in increased surface roughness, a higher density of oxygen-containing functional groups, and enhanced physicochemical heterogeneity [8]. First, the release of low-molecular-weight organic acids during weathering may alter soil pH, increase H^+^ concentrations, and consequently modify electrostatic adsorption processes and soil redox conditions [9]. Second, as exogenous particulate materials, MPs can interfere with the formation and stability of soil macroaggregates, thereby altering local soil physical structure [10]. Furthermore, because MPs themselves are carbon-containing materials, their accumulation may influence soil carbon cycling by altering the sequestration of soil organic matter, stimulating microbial activity, and creating microbial hotspots [11]. These physicochemical alterations collectively suggest that MPs may substantially reshape the soil microenvironment, thereby indirectly modulating the transport, mobility, and fate of co-existing contaminants in agricultural soils.

Heavy metals (HMs) are widespread contaminants in agricultural soils. Historically, the application of metal-containing fertilizers and pesticides, as well as irrigation with contaminated water, has been regarded as the primary input pathways of HMs in croplands [12,13]. In recent years, however, evidence emerging from MP-contaminated soils suggests that MPs accumulation may further complicate HM environmental behavior. Beyond acting as an environmental matrix, plastics themselves can contribute to HM inputs through the leaching of metal additives incorporated during plastic manufacturing [14,15]. More importantly, their small size gives endow MPs with high specific surface areas and abundant adsorption sites [16]. Environmental aging further increases their surface roughness and electronegativity, substantially enhancing their capacity to interact with HMs through electrostatic attraction, surface complexation, and cation bridging [17]. Consequently, MPs have been hypothesized to serve as vectors that for adsorbed HMs, potentially modulating contaminant migration across soil profiles and subsequent biological exposure [18].

Controlled experiments have demonstrated that MPs can increase HM concentrations and alter their mobility in soils [19,20]. However, interactions between MPs and HMs in natural agricultural soils are considerably more complex than those observed under controlled laboratory conditions. Ongoing aging continuously modifies MP surface properties, while soil characteristics such as pH, cation exchange capacity (CEC), and soil organic matter (SOM) interact with external anthropogenic and environmental drivers (including plowing, fertilization, freeze–thaw cycles and acid rain) to jointly regulate HM adsorption, desorption, and transformation [9,21,22,23]. Consequently, the environmental behavior of MP–HM co-contamination is governed by multiple interacting processes rather than direct particle–metal interactions alone. Furthermore, despite providing fundamental insights into MP–HM interactive mechanisms under controlled conditions, laboratory studies relying on homogenized agricultural soils [9,10,20,22,24,25,26,27] and cannot fully capture the spatial heterogeneity induced by long-term agricultural management. Although several field investigations have reported elevated concentrations of free HM ions in agricultural soils with prolonged plastic-film application histories [28,29,30], the spatial distribution and depth-dependent profiles of such co-contamination remain poorly understood [9,22,23,24,30]. Therefore, systematic field-based investigations are indispensable to elucidate the real-world co-occurrence patterns and associated risks of MP–HM co-contamination under realistic agricultural conditions [9,10,20,23,24,25,26,27,28,31], particularly regarding whether MP accumulation aligns with shifts in the chemical speciation and vertical redistribution of legacy HMs under realistic agricultural conditions [31].

Based on this background, this research focuses on the Anning Valley to explore the complex MP occurrence and its associations with HM. Anning Valley is a representative agricultural region in southwestern China featured with relatively elevated geogenic background levels of heavy metals [32,33]. Combined with intensive plastic use in greenhouse canopy-film farmland, this region presents a high likelihood of MP-HM co-contamination [33,34]. A spatiotemporal sequence of plots with varying cultivation years (1~8 years) was established and profile soil samples were systematically collected from three depths (Layer A: 0~10 cm, B: 10~20 cm, C: 20~40 cm) to comprehensively investigate MP abundance, physicochemical characteristics, HM loads, and HM chemical speciation.

This study addresses three specific research questions:

(1) What are the spatiotemporal dynamics of MP accumulation and weathering in greenhouse farmlands of the Anning Valley? (2) Do HM concentrations, vertical distributions, and chemical speciation exhibit patterns consistent with MP accumulation in these soils? (3) Are MP–HM co-contamination patterns spatially homogeneous, or do they vary across soil depths and among different metal elements?

We hypothesized that MP accumulation would align with localized HM enrichment and shifts in HM chemical partitioning; however, these co-contamination patterns would not be uniform, but rather exhibit strong spatial dependence and element-specific differences. This study aims to provide field-based observational evidence for understanding the long-term evolution of soil MP pollution and the heterogeneous characteristics of MP–HM co-contamination in greenhouse agricultural systems.

## 2. Methodology

### 2.1. Study Area and Sample Collection

The study area is located within the Anning River Valley y (26°36′–28°56′ N, 101°47′–102°44′ E), situated in the geomorphic transition zone between the Yunnan–Guizhou Plateau, the Qinghai–Tibet Plateau, and the Sichuan Basin (Figure 1A). The region is dominated primarily by Entisols and Spodosols, with soil textures ranging from medium-fine to coarse fractions [34,35]. Agriculture serves as the primary industry in the Anning River Basin, making it a critical grain production hub in Sichuan Province [35]. Influenced by the southwest monsoon, the region experiences considerable climatic variability [36], leading to the widespread adoption of greenhouse systems that regulate temperature, humidity, and light. Our sampling sites were concentrated west of Xichang City, where extensive contiguous greenhouse grape orchards are distributed across the central part of the Anning River Plain (Figure 1B) [37]. Xichang is renowned as the “Home of Late-Maturing Grapes in China” because of its favorable agricultural conditions, including optimal light, temperature, water, and soil resources. However, while protective greenhouse canopy films are widely deployed to shield fruit from intense solar radiation and sunscald, long-term exposure to high UV levels—combined with inadequate end-of-life management—causes film embrittlement and fragmentation. This process continuously feeds plastic debris into the soil matrix, serving as a primary ongoing source of agricultural MPs [37,38,39]. In addition, Xichang has a historical association with non-ferrous metal smelting, and previous studies have reported relatively elevated regional background levels of several heavy metals [32,33]. Against this geochemical background, intensive greenhouse agriculture and extensive plastic use may create conditions conducive to the co-occurrence of MPs and HMs in agricultural soils, underscoring to the need to characterize their spatial distribution and co-occurrence patterns.

As shown in Figure 1C, one representative plot was selected for each of the four cultivation-year categories based on greenhouse film usage duration: 1~2 years (GY0), 3~4 years (GY1), 5~6 years (GY2), and 7~8 years (GY3). These plots were selected from a contiguous vineyard area subject to unified management to minimize variability in baseline soil properties and agronomic practices, thereby allowing a preliminary, exploratory comparison across progressive cultivation stages. Given that MPs in farmland soils are primarily enriched within the upper 0–30 cm horizon [40], soil profile samples were systematically collected from three depth intervals (Layer A: 0~10 cm, Layer B: 10~20 cm, and Layer C: 20~40 cm) at each plot to elucidate the vertical distribution patterns of MPs. For each plot and depth interval (12 plot–depth combinations in total), three independent composite samples were collected as replicates. Each composite sample was prepared by thoroughly homogenizing five spatial subsamples collected following an S-shaped sampling pattern.

Sampling was conducted in April 2025 using a stainless-steel spade and shovel, with precautions taken to prevent plastic contamination. Before lab experiment, soil intended for MP analysis was stored in pre-cleaned aluminum containers, whereas soil for HMs and other physicochemical properties was stored in sealed polyethylene bags at ambient temperature. Soil samples were first air-dried for approximately 10 days. Subsequently, they were gently ground in an agate mortar and passed through a 2 mm stainless-steel sieve.

### 2.2. Sample Processing and Chemical Analysis

For each sample, MPs were extracted from a standardized 30 g aliquot of air-dried soil based on density separation and chemical digestion methods with modifications [41]. Briefly, MP particles larger than 20 μm were targeted. Density flotation was performed using a ZnCl_2_ solution (density: 1.55 g/mL), followed by digestion with Fenton’s reagent (30% H_2_O_2_ + 0.05 M FeSO_4_) to remove organic impurities. The extracted MPs were then identified using micro-Raman spectroscopy, with particle counts and morphological features recorded. Details of operation are summarized in Table S1.

The total concentrations of cadmium (Cd), chromium (Cr), nickel (Ni), lead (Pb), zinc (Zn), and copper (Cu) were determined by Inductively Coupled Plasma Mass Spectrometry (ICP-MS, Agilent 7900, Agilent Technologies, Santa Clara, CA, USA). All procedures strictly followed the Chinese national standard (HJ 803–2016) [42]. The modified Community Bureau of Reference (BCR) sequential extraction protocol was applied exclusively to soil samples from the A layer (0–10 cm), where the highest MP abundance was observed [43]. This method partitions metals into four operationally defined fractions based on binding strength and environmental mobility: F1 (Acid-soluble/Exchangeable), Metals with immediate bioavailability and high mobility; F2 (Reducible), Metals bound to Fe/Mn oxides, mobile under reducing conditions; F3 (Oxidizable), Metals associated with organic matter and sulfides, released slowly under oxidizing conditions; F4 (Residual), Metals incorporated within the crystalline structure of silicate minerals, possessing minimal environmental mobility under normal conditions [44]. Description of analytical methods and testing procedures for soil physicochemical properties are shown in Table S1.

### 2.3. Evaluation of Heavy Metal Contamination

#### 2.3.1. Pollution Assessment

To comprehensively evaluate pollution by heavy metals in MP-bearing farmland, we employed four indices: the Single-factor Pollution Index ($P_{i}$), Nemerow Integrated Pollution Index (*NPI*), geo-accumulation index ($I_{geo}$) and potential ecological risk index (PERI), calculated according to the following equations [45,46,47].

$$P_{i}=\frac{C_{i}}{S_{i}}$$

where $C_{i}$ is the measured concentration of heavy metal *i*, and $S_{i}$ is the corresponding reference value. In this study, the screening values for agricultural soil specified in the Risk Intervention Values for Agricultural Land in China (GB 15618-2018) [48] was used as reference values.

$$NPI=\sqrt{\frac{{P_{imax}}^{2}+{P_{iave}}^{2}}{2}}$$

where NPI denotes the integrated pollution index, $P_{imax}$ is the highest $P_{i}$ value among assessed heavy metals, and $P_{iave}$ is the mean $P_{i}$ value among assessed heavy metals.

$$E_{r}^{i}=T_{r}^{i}\frac{C_{i}}{C_{n}^{i}}$$

$$PERI=\sum_{i=1}^{n}E_{r}^{i}$$

where $E_{r}^{i}$ denote potential ecological risk of single heavy metal element, and PERI represents the integrated potential ecological risk of various heavy metals. $T_{r}^{i}$ is the toxicity-response coefficient of heavy metals [Cu(5), Pb(5), Zn(1), Cd(30), Cr(2), and Ni(5)] [49], and $C_{n}^{i}$ refers to the threshold for element i and Chinese standard (GB 15618-2018) was used in this study for these values.

$$I_{geo}=\log_{2}\left(C_{i}/kB_{i}\right)$$

where $C_{i}$ is the soil concentration of heavy metal *i*, and $B_{i}$ is the local background value of soil heavy metal *i* [50], and *k* is a variation factor accounting for lithogenic variations (generally taken as 1.5) [50].

The contamination classification criteria for the respective heavy metal risk indices are detailed in Table S2.

#### 2.3.2. Health Risk Assessment

Health risk was assessed using model recommended by the United States Environmental Protection Agency (USEPA). Accumulative non-carcinogenic risk and carcinogenic risks were quantified as the Hazard Index (*HI*) and the Carcinogenic Risk (*CR*), respectively. Three exposure pathways—ingestion, inhalation, and dermal contact—were considered [30]. The exposure formula used for this evaluation are expressed as follows:

$${ADD}_{ingestion}=\frac{C_{S}\times IngR\times EF\times ED\times 10^{-6}\;}{BW\times AT}$$

$${ADD}_{inhale}=\frac{C_{S}\times InhR\times EF\times ED}{PEF\times AT\times BW}$$

$${ADD}_{dermal}=\frac{C_{S}\times SA\times AF\times ABS\times EF\times ED\times 10^{-6}}{BW\times AT}$$

$$HI=\sum \left(\frac{{ADD}_{ij}}{{RfD}_{ij}}\right)$$

$$CR=\sum \left({ADD}_{ij}\times {SF}_{ij}\right)$$

where ${ADD}_{ingesiton}$, ${ADD}_{inhale}$, and ${ADD}_{dermal}$ denote the estimated average daily doses through soil ingestion, soil inhalation, and dermal contact with soil, respectively; $C_{S}$ represents the total measured concentration of heavy metal *i* in soil. CR represents the carcinogenic risk; a value below 1.0 × 10^−6^ indicates a negligible cancer risk, whereas a CR exceeding 1.0 × 10^−4^ implies a significant increase in the probability of developing cancer. HI is the sum of hazard quotients (HQs) for all metals and exposure routes, where HI < 1 suggests that the non-carcinogenic health risk from heavy metals in soil is negligible. $C_{i}$ represents the concentration of heavy metals in soil [mg·kg^−1^]. Parameters were systematically synthesized from relevant peer-reviewed studies and are provided in Tables S3 and S4. Specifically, fundamental exposure models and default intake equations were adopted from the USEPA Exposure Factors Handbook, whereas key physical and physiological parameters—including body weight (BW) and Inhalation rate of soil (InhR)—were tailored to Chinese localized scenarios by referencing the Bulletin of National Soil Pollution Survey [51,52,53,54]. Because children are more susceptible to health risks associated with heavy metals in soil and are generally more sensitive to environmental pollutants [51], health risks were calculated for children as the target population. Given that Cr speciation was not directly measured in this study, health risk for Cr were evaluated under upper-bound screening assumption that all measured Cr exists as Cr (VI), providing a conservative scenario.

### 2.4. Quality Control

To minimize contamination during sampling and laboratory processing, plastic materials were avoided whenever possible. All glassware was rinsed at least three times with purified water before use, and all reagents were filtered through a 10 μm stainless-steel sieve. Samples were handled in covered glass containers, and laboratory personnel wore cotton lab coats throughout sample preparation and analysis. Procedural blanks were processed alongside each batch of samples. No microplastics were detected in the procedural blanks. All analytical results were quality-controlled and corrected against the corresponding blank controls where appropriate. Recovery experiments were performed using polymer-spiked samples, yielding particle recoveries of 86.7–100.0%. Because MPs and HMs are ubiquitous throughout the study area, a true field blank could not be established. Consequently, the plot with the shortest cultivation history (GY0, 1–2 years) was designated as the baseline scenario for temporal comparisons.

### 2.5. Statistical Analysis

Prior to statistical analysis, data were assessed for normality using the Shapiro–Wilk test and for homogeneity of variances using Levene’s test. The assumptions of parametric analyses were satisfied without data transformation. Differences among cultivation stages and soil layers were evaluated using one-way or two-way analysis of variance (ANOVA), as appropriate. Post hoc multiple comparisons were performed using Tukey’s honestly significant difference (HSD) test. The chi-square test was applied to compare categorical variables, specifically the distribution of MPs across shape, color, and size categories. Pearson correlation analysis was performed to evaluate relationships among MPs, HMs, and soil physicochemical properties. Principal component analysis (PCA) was conducted to reduce data dimensionality and elucidate the primary multivariate relationships. The significance level was set at α = 0.05. Statistical analyses were performed in SPSS version 24. Microsoft Excel l (Microsoft 365) and Origin 2024 were used for data processing and figure rendering.

## 3. Results and Discussion

### 3.1. Progressive Surface Accumulation of MPs

Microplastics were detected in all soil samples (Figure 2), exhibiting a mean MP abundance of 1663.92 ± 1336.96 n/kg (range: 389.81~5045.65 n/kg) across the entire soil profile This abundance level is comparable to those reported in agricultural soils under intensive plasticulture in previous studies [41]. Overall, MP abundance displayed a clear upward trend with prolonged cultivation: the late-stage plot (GY3, 3373.19 n/kg) contained significantly higher MP levels than the initial -baseline plot (GY0, 758.53 n/kg). The Anning Valley is characterized by intense solar radiation, large diurnal temperature variations, and strong winds. These climatic conditions necessitate extensive application of protective greenhouse canopy films; subsequently, prolonged exposure under intense ultraviolet (UV) irradiation [55], mechanical forces [56] and thermal fluctuations [38] accelerates the weathering and fragmentation of agricultural plastic residues.

The topsoil was identified as the primary accumulation zone, where MPs were significantly enriched. MP abundance increased earliest and most rapidly in Layer A, ultimately establishing a pronounced vertical gradient between the topsoil and the deep layer (2462.25 vs. 951.25 n/kg on average). This depth-dependent accumulation pattern suggests that MP inputs were primarily controlled by surface-derived sources, including the application and weathering of agricultural plastic film, rather than by indigenous sub-surface processes. Although vertical transport can occur via preferential flow paths or soil cracks [57], our results revealed that MP abundance dropped markedly in the deeper soil layer (20–40 cm). This disparity suggests that even when MPs migrate downward, the absence of surface environmental drivers—such as direct UV exposure, thermal fluctuations, and mechanical tillage—prevents their further fragmentation into smaller particles. Because subsoil MP breakdown relies primarily on slow microbial colonization and slow biodegradation [17], processes constrained by the dry microclimate of the Anning Valley [58], subsoil MPs do not undergo the rapid numerical multiplication observed in the topsoil. Nonetheless, their continuous detection throughout the 0–40 cm soil profile shows that the soil matrix retards, rather than precludes, their downward transport. This pattern may have important ecological implications because the upper soil horizon hosts most plant roots and microbial activity and is therefore closely linked to crop productivity and food safety [11].

### 3.2. The Characteristics of Weathered MPs

Because the environmental behavior and ecological effects of MPs depend not only on their abundance but also on their physicochemical traits [59], optical microscopy was further employed to capture real morphological characteristics and examine the temporal changes across cultivation years. As illustrated by the representative observations in Figure 3 agricultural MPs underwent clear structural degradation, fragmenting into micrometer-scale particles with translucent, highly irregular configurations and surface flaws. These physical signatures reflect progressive environmental weathering and fragmentation of MPs under long-term field exposure [19,22].

Figure 4 illustrates both the profile-averaged and plot-level compositional characteristics of MP types and morphologies. As presented in Figure 4A, the predominant polymer types identified were polyethylene (PE, 31.43% in average) followed by ethylene–propylene copolymer (E/P), polyamide (PA), and polypropylene (PP), each constituting over 15% of the total. This polymer composition strongly aligns with the widespread application of protective greenhouse canopy films, with secondary contributions potentially arising from auxiliary agricultural plastics, such as ropes, fertilizer bags, and irrigation piping components [28]. Notably, PA exhibited a notably higher proportion specifically in GY3 (the pink band in Figure 4A), which may reflect differences in polymer durability and the cumulative history of plastic inputs [17]. This compositional shift carries long-term ecological concern, as weathering-resistant materials such as PA may exhibit distinct environmental residence and toxicological profiles [60].

The shape composition changed significantly across cultivation years (*p* < 0.05, Figure 4B). Fragments were the predominant morphology overall (49.36%), though their relative proportion decreased from over 60% in early stages (GY0 and GY1) to 38.51% in GY3 (the yellow segment). Conversely, the proportion of micro-sized pellets (the dark blue segment in Figure 4B) increased from 8.00% to over 35.00%, consistent with continuous mechanical abrasion and disintegration of larger plastic debris under field exposure. A significant shift in color composition was also observed across cultivation years (*p* < 0.05, Figure 4D), characterized by a notable enrichment of MPs within the yellow-tan color spectrum (reaching 61.53% in GY3 vs. 45.40% in GY0, the yellow segment). This chromophoric shift aligns with UV-induced photo-oxidation, chromophore formation [55], and pigment photodegradation or oxidative leaching [61].

As shown in Figure 4C, the size distribution was dominated by small-to-medium particles across plots, with <100 µm and <500 µm fraction accounting for 17.65% and 79.31% on average, respectively. The ongoing fragmentation into finer micro- and nanoplastics presents elevated risks of cellular internalization and trophic transfer along food webs [28,62]. Intriguingly, the GY3 stage exhibited a higher proportion of medium-to-large particles, where the [250, 500) µm (represented by pink segment in Figure 4C) and [500, 1000) µm size classes (dark orange segment) expanded to 36.5% and 18.3%, respectively. This shift suggests that the periodic input of fresh, macro-sized plastic residues can temporarily outpace in situ fragmentation rate under prolonged greenhouse management.

### 3.3. Reversed Vertical Distribution During HMs Co-Accumulation

Soil HM concentrations followed a descending order: Zinc (Zn, 56.67~510.02 mg/kg) > Chromium (Cr, 59.98~112.90 mg/kg) > Nickel (Ni, 25.34~47.97 mg/kg) > Lead (Pb, 19.80~57.85 mg/kg) > Copper (Cu, 19.67~30.59 mg/kg) > Cadmium (Cd, 0.18~10.86 mg/kg) (Table 1). As shown in Figure 5, long-term agricultural cultivation was accompanied by pronounced HM accumulation, showing strong spatial alignment with MP-enriched soil horizons and plots, though the enrichment magnitude differed markedly among elements. Unlike Cr, Ni, Cu, and Pb—which remained relatively stable—Cd and Zn exhibited extreme spatial variability (CV > 70%), with their mean concentrations in GY3 expanding to 3.54-fold and 25.97-fold of baseline levels in GY0, respectively.

As presented in Figure 5, long-term HM enrichment was accompanied by a pronounced vertical profile redistribution. In the baseline stage (GY0), HM concentrations in the deep layer (Layer C) were comparable to (as in Figure 5A–C for Cd, Cu and Zn) or higher than those in the surface layer (as in Figure 5D–F for Ni, Pb and Cr). With increasing years of greenhouse canopy-film cultivation, however, the accumulation rate accelerated preferentially in the topsoil. By the GY3 stage, topsoil had emerged as the dominant accumulation zone For Cd, Cu, Zn and Pb, forming a clear Layer A > Layer B > Layer C concentration gradient. This progressive profile inversion demonstrates that long-term agricultural inputs are heavily topsoil-anchored rather than uniformly throughout the soil profile.

### 3.4. Patterns of HM Partitioning Toward Potentially Mobile Fractions

In topsoil subject to MP accumulation, HM chemical speciation evolved markedly over cultivation time, revealing distinct element-specific redistribution patterns. As shown in Figure 6A–F, speciation profiles shifted noticeably from relatively stable pools (F3/F4) toward potentially mobile pools (F1/F2) for most metals, including Cd, Pb, Zn, and Ni. This trend is evidenced by the dark and light red segments (representing F1 and F2, respectively) extending along the positive axis in Figure 6G). Previous studies have demonstrated that soil Cr and Cu are governed more strongly by inherent geochemical properties than by anthropogenic factors [63]. Consistent with this notion, their mobile fractions (F1 + F2) did not increase significantly in our samples; instead, internal redistributions occurred primarily within their stable pools (F3 and F4): the residual fraction (F4) of Cr increased by 7 percentage points, while the oxidizable fraction (F3) of Cu expanded by 20 percentage points alongside a concurrent 3 percentage point rise in F4 (*p* < 0.05).

Among all examined elements, Cd exhibited the strongest geochemical activation over time. Its active fractions—comprising both the acid-soluble (F1) and reducible (F2) pools—accounted for a major proportion in the GY0 plot (71.14% combined) and continuously escalated to 93.02% in the GY3 plot, consistent with recent findings on MP–HM interactions [28]. Meanwhile, Pb, Zn, and Ni displayed a significant increase in the reducible fraction (F2) (*p* < 0.05) along the MP gradient, by 15.99, 11.68, and 6.96 percentage points, respectively. Co-occurring with MP accumulation, this shared response suggests that these metals preferentially partition into Fe/Mn oxides, underscoring the role of these oxides as a key geochemical phase mediating MP-HM co-occurrence.

Compared with pristine soil minerals—which intrinsically possess a significantly greater specific surface area, higher porosity, and denser array of charged surfaces [64]—MPs have been reported to exhibit weaker binding affinities and higher metal desorption potential [65]. Consequently, we hypothesized that the incorporation of MPs effectively “dilute” the overall sorption capacity of the solid phase, where adsorbed metal ions more readily desorb into the soil solution, thereby contributing to the observed increase in HM bioavailability and mobility.

### 3.5. Accelerated HM Risk Indicated by Multiple Indices

Multiple pollution and ecological risk indices were integrated to evaluate the environmental consequences of the observed HM enrichment. Specifically, *P_i_* and *NPI* assess contamination relative to regulatory thresholds, *I_geo_* evaluates enrichment relative to local geochemical background values, whereas *PERI* additionally incorporates the toxic-response coefficient of each element.

On average, the *NPI* across all samples reached 4.38, indicating a state of heavy overall pollution, while the mean *PERI* reached 185.8, corresponding to a moderate ecological risk (detailed evaluation results are presented in Figure 7A,B). Risk contribution analysis (Figure 7C,D) identified Cd as the primary contributor to HM risk in the study area, accounting for 81.27–96.25% of the *PERI* across plots (averaged over soil depths) and 10.74–81.91% of the NPI, aligning with national patterns reported for Chinese agricultural soils [66]. The clear dominance of Cd in *PERI* reflects both its exceptionally high toxicity-response coefficient (30) and its elevated concentrations. Coupled with the high proportion of acid-soluble Cd (F1, 30–50%, Figure 6D), Cd represents the priority contaminant in this system. Similarly, *I_geo_* confirmed severe Cd enrichment—exceeding the “heavy pollution” threshold (*I_geo_* > 3) at all sites (ranging from 7.60 to 8.80)—while other heavy metals remained within clean to moderate polluted levels (Figure 7E).

Rather than increasing linearly with cultivation years, HM risk appeared to escalate after prolonged plastic-film cultivation, suggesting a threshold-like escalation in contamination. As illustrated in Figure 7A,B,E, high values were concentrated in Layer A and Layer B of plots with longer cultivation histories, mirroring the spatiotemporal patterns of MPs occurrence. The GY0 and GY1 were generally clean, whereas marked risk deterioration occurred from GY2 onward, escalating sharply in GY3. Vertically, Layer C consistently showed lower pollution levels; however, even the deepest horizon reached severe Cd pollution (Cd *P_i_* = 3.41) and alert-level overall pollution (*NPI* = 2.48) in GY3.

Cross-index evaluation provided critical insights into distinguishing anthropogenic contamination from natural geological background. Zn displayed a moderate increase in *P_i_* (reaching 1.96 in GY3) whereas its *I_geo_* values remained persistently negative throughout, suggesting only limited enrichment relative to the naturally high regional background. Conversely, Ni exhibited positive *I_geo_* values alongside consistently low *P_i_* and *PERI* values, indicating that its elevated concentrations mainly reflected the naturally metal-rich parent material of the Anning Valley rather than anthropogenic pollution. Integrating multiple indices therefore enables discrimination between background-controlled enrichment and anthropogenic accumulation requiring regulatory action.

### 3.6. Acceptable Non-Carcinogenic and Persisting Carcinogenic Risk

While environmental indices quantify environmental contamination, they do not directly reflect potential risks to human receptors. Therefore, a human health risk assessment targeting children—a vulnerable subpopulation with heightened susceptibility—was conducted based on the USEPA model.

As shown in Figure 8A, total HI values across all sampling sites were well below the safety threshold of 1.0, ranging from 0.35 to 0.68, indicating that non-carcinogenic health risk from soil HMs in the study area is negligible. In contrast, total CR values across sampling sites ranged from 1.72 × 10^−4^ to 8.56 × 10^−4^, all exceeding the significant-risk threshold of 1 × 10^−4^ (Figure 8B). Soil ingestion was identified as the dominant exposure pathway, accounting for 92.7% and 99.8% to the total HI and CR, respectively, consistent with previous studies [67]. This scenario is of particular public health concern because children engage in frequent outdoor soil contact and exhibit frequent hand-to-mouth behavior, substantially increasing incidental soil ingestion [68]. Furthermore, the spatial pattern of both HI and CR closely mirrored the distribution of MPs and HMs, with the peak values consistently localized in topsoil horizons in prolonged facility cultivation (the upper left segments in Figure 8A,B).

Element-specific analysis revealed contrasting drivers for non-carcinogenic versus carcinogenic risks. As shown in Figure 8B, Cr contributed >60% to HI in all plots, primarily due to its conservative RfD combined with elevated concentrations. However, the overall risk reported here should be interpreted as conservative, upper-bound screening estimates. BCR extraction revealed Cr predominantly in stable fractions (F4 ~50%, F3 ~44.5%, Figure 6A), while the USEPA model assumes complete bioavailability based on total concentrations [67]. Furthermore, Cr was evaluated based on total concentrations rather than $Cr\left(VI\right)$, likely overestimating its actual non-carcinogenic risk. In contrast, Cd non-carcinogenic risk exhibited an 11-fold escalation from GY0 to GY3, coinciding with a high acid-soluble fraction (F1, 30–50%), which suggests its total concentration better reflects true environmental bioavailability.

As in Figure 8C, CR was predominantly driven by Ni and Cd, whose combined contribution exceeded 99.9%. The CR attributed to Ni remained relatively stable (1.55 × 10^−4^–2.37 × 10^−4^), confirming that its carcinogenic risk mainly reflects the naturally metal-rich regional background rather than progressive anthropogenic enrichment. In contrast, Cd exhibited a marked increase throughout cultivation timeline, with its contribution to topsoil CR surging from 11% at GY0 to 74% at GY3, translating to a 32-fold increase in absolute risk.

Collectively, the health risk assessment complements the environmental pollution indices by demonstrating that HM enrichment does not necessarily translate into proportionally greater human health risk. Instead, the health implications depend on both exposure pathways and metal bioavailability, which underscores the need to integrate chemical speciation with conventional risk assessment frameworks.

### 3.7. Spatial and Elemental Dependence of MP–HM Association Patterns

It is important to acknowledge that the observed parallel increases in MP abundance and HM concentrations over cultivation years do not inherently imply a direct causal link. Long-term greenhouse farming involves continuous applications of plastic canopy films, commercial fertilizers, pesticides, and irrigation water, which collectively act as persistent sources for both MPs and heavy metals (e.g., Cd and Zn from phosphate fertilizers or plastic additives). These shared or parallel input pathways could independently drive the accumulation of both contaminant classes over time. Nevertheless, the correlation analyses presented here serve two exploratory purposes: (1) to reveal the co-linear temporal trajectories resulting from intensive long-term agricultural inputs, and (2) to identify patterns that may point toward potential secondary interactions between aged MPs and metal ions within the soil–matrix interactions that warrant further mechanistic investigation.

Multivariate analyses were conducted to evaluate the relationships among MPs, HMs, and other physicochemical properties. As presented by PCA results in Figure 9A, the first two principal components (PC1 and PC2) explained 53.3% and 19.8% of the total variance across all soil samples, respectively. PC1 captured the primary co-occurrence pattern, with higher PC1 scores corresponding to elevated concentrations of Cu, Zn, Cd, Pb, MPs, SOC, and silt content, together with lower pH and sand content. In contrast, PC2 primarily reflected variations in Cr, Ni, and clay content. Layer-specific PCA revealed distinct association patterns across soil depths (Figure 9(B1–B3)). Topsoil (Layer A) samples clustered predominantly in the high-PC1 region, displaying strong linkages among MPs, Cu, Zn, Cd, Pb, and SOC. In contrast, deep-layer samples (Layer C) shifted toward the high-PC2 region, where MPs-HM distribution became “decoupled”, reverting to association with natural factors like clay content and parent material. From Layer A to Layer C, the spatial coupling between MPs and several HMs became progressively weaker, whereas HM associations with clay became relatively stronger.

Figure 10A illustrates the correlation analysis results between total heavy metal concentrations and other soil environmental variables, revealing metal-specific patterns where the six HMs separated into two distinct groups: (1) Cu, Zn, Cd, and Pb displayed significant positive correlations with SOC and all MP size fractions larger than 50 μm, significant negative correlations with soil pH (except for Cu); (2) in contrast, Cr and Ni demonstrated a markedly different pattern showing significant correlations with soil texture, but only weak correlations with SOC, pH and MPs (confined to the 100–250 μm and 500–1000 μm size fractions).

HMs in topsoil (Layer A) were further fractionated into chemical speciation pools (F1–F4) to evaluate their specific associations with environmental variables (Figure 10(B1–B6)): (1) For Pb, Zn, Cd, and Cu, the correlation patterns of mobile fractions (F1 and F2) exhibited correlation profiles resembling those of total concentrations—displaying strong positive associations with MP abundance, SOC, and fine soil particles (silt), alongside inverse trends with pH and coarse fractions (sand). Element-specific correlation detailed descriptions of the key correlations are presented in Table S5. (2) for Cr and Ni, chemical fractions showed no significant correlations with MP abundance, remaining instead more strongly associated with soil texture variables.

The MP–HM association observed here likely reflects shared agricultural drivers and localized matrix interactions, rather than a simple unidirectional effect of MPs on HM behavior. Previous studies have shown that MPs can be associated with HM enrichment [20], whereas soil pH, organic matter, and particle-size distribution are also well-recognized factors governing HM behavior [69]. Rather than acting entirely independently, the observed MP–HM coupling may stem from the capacity of MPs to modify these governing soil factors. In natural soils, MPs may interact with these environmental factors by releasing dissolved organic carbon [11] and promoting localized acidification [8]. Our correlation results provide several observations consistent with this possibility.

Soil texture is an important environmental factor governing the retention, redistribution, and vertical transport of HMs by influencing specific surface area, reactive mineral surfaces, pore structure, and water movement in soils [70]. Traditionally, clay minerals are considered helpful in reducing HM migration due to strong retention capacity [69,70,71]. This conventional paradigm remains partly valid across cultivation durations, as finer fractions of silt and clay expanded alongside progressive HM accumulation over time; however, distinct depth-dependent nuances emerged. Although principal component analysis (PCA) revealed that HM concentrations in Layer C (where MP abundance was minimal) remained strongly aligned with clay content, topsoil horizons (Layers A and B) exhibited concurrent enrichment of HMs and coarser sand fractions (Figure S1C–E) which intrinsically demonstrate limited native adsorption capacity [72]. As a result, clay (the finest fraction) maintained significant positive correlations only with Cr and Ni—two metals weakly associated with MPs—while its relationships with the remaining four MP-correlated metals (Cu, Zn, Cd, and Pb) became non-significant. Instead, these four metals shifted toward stronger associations with silt and coarser MPs (>50 μm). Given that MPs can become incorporated into larger soil aggregates [10,73], these parallel observations raise the speculative interpretation that the presence of MPs within coarser soil fractions may reshape the texture-controlled partitioning of heavy metals, shifting their apparent geochemical association away from fine clay minerals toward MP-enriched coarser fractions.

The multivariate analyses also revealed that MP–HM associations differed substantially among individual metals. Unlike Cu, Zn, Cd, and Pb which closely linked to MPs, Cr and Ni remained more closely associated with soil texture and exhibited comparatively limited changes in chemical speciation. Plastic materials selectively change soil metals distributions as they represent an additional source through the gradual release of metallic additives, including Cd and Zn stabilizers or Pb- and Cu-containing catalysts [15]. Although not directly measured in this study, the potential release of such additives could contribute to the elevated concentration in soil. Previous studies have shown that adsorption affinities between MPs and individual metals can differ substantially, providing a plausible basis for the element-specific co-variation patterns observed in our field samples. For example, Cu^2+^ exhibits stronger adsorption onto MPs than Ni^2+^ [74]. Similarly, Cd shows high mobility even under relatively low MP concentrations, whereas comparable effects on Cr required much higher MP loadings [75]. In the present study, as cultivation years increased, MP exhibited progressively stronger signs of weathering, process known to foster the formation of oxygen-containing functional groups (e.g., –COOH and –OH) [17,21]. These temporal co-variations raise the possibility that the aged MPs may further contribute to the element-specific association patterns observed here. Nevertheless, this remains a hypothesis that requires direct characterization of functional groups on field-aged MPs and controlled sorption experiments to confirm.

## 4. Conclusions

This study shifts attention beyond the immediate agronomic benefits of plastic mulching to its long-term legacy as a persistent source of soil microplastics. It also extends the scope from single-contaminant frameworks to the spatial co-occurrence of an emerging contaminant (MPs) with legacy HMs in greenhouse agricultural soils. Our field observations demonstrate that MPs accumulated and enriched in topsoil progressively with cultivation time, resulting in pronounced surface enrichment and vertically stratified pollution risks (A > B > C). Crucially, in topsoil the accompanying heavy metal dynamics involved not only an overall elevation in total concentrations but also an internal activation of chemical mobility. A major finding of this study is that MP–HM associations exhibited both spatial and element-specific patterns. Higher MP abundance consistently coincided with higher HM concentrations, larger proportions of potentially mobile fractions, lower pH, higher SOC, and shifts in texture-controlled metal partitioning. However, these associations weakened along the vertical profile, becoming decoupled in deeper layers horizons where geogenic controls predominated. Furthermore, MP–HM associations also differed markedly among metals: Cu, Zn, Cd, and Pb displayed strong spatial linkages with MPs, whereas Cr and Ni remained overwhelmingly governed by clay mineral distribution and regional lithogenic background. Notably, Cd exhibited the greatest enrichment, the largest increase in potentially mobile fractions, and progressively greater contributions to HM pollution and health risks. Overall, these findings provide robust field-scale evidence that MP–HM co-contamination is spatially heterogeneous and metal-specific rather than uniform. Future studies integrating controlled experiments and mechanistic investigations are needed to determine the processes underlying these association patterns and to support soil quality management in agricultural systems under long-term plastic use.

## 5. Limitation

Sampling Scale: Only one representative plot was selected for each cultivation-year category, with the three replicates (*n* = 3) representing within-plot subsamples rather than independent field replicates. Consequently, the statistical analyses (e.g., ANOVA) and the observed patterns are primarily used for exploratory description and hypothesis generation, rather than for definitive inference of causal relationships or temporal trends. The results should be interpreted as indicative field evidence from a typical management sequence.

Speciation Depth: Chemical speciation via BCR sequential extraction was conducted exclusively on Layer A soils. While Layer A represents the primary zone of microplastic accumulation and active agrochemical management, the lack of speciation data for deeper soil horizons limits our ability to fully resolve the mechanistic drivers of vertical metal redistribution down the profile. Future investigations incorporating multi-depth speciation and column leaching assays are needed to confirm the vertical migration behaviors of specific metal fractions.

Vector Verification: While our correlation analyses suggest potential associations between MPs and HMs, we did not directly measure metal loads on MP surfaces nor conduct desorption experiments to confirm the release or uptake of metals by MPs. Therefore, any discussion of MPs acting as ‘vectors’ for metal transport remains speculative and requires direct experimental verification.

## Figures and Tables

**Figure 1:**
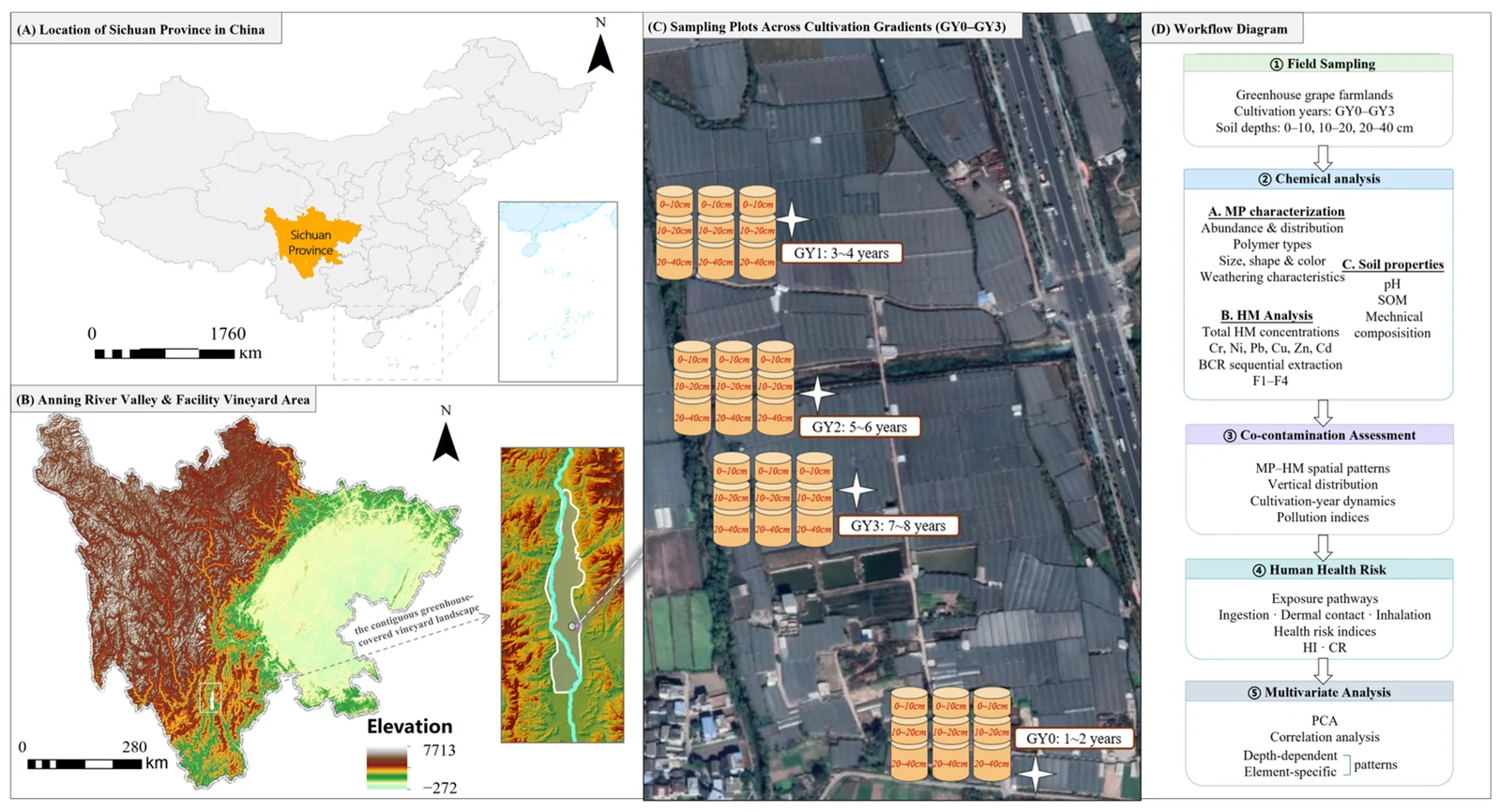
Study area information and workflow diagram. (**A**) Geographic location and boundaries of Sichuan Province within China. (**B**) Location of the Anning River Valley Plain in Sichuan Province, with an inset map depicting the contiguous facility canopy-covered vineyard landscape in Xichang City. (**C**) Spatial distribution of the four sampling plots across continuous greenhouse film cultivation duration gradients: GY0 (1–2 years), GY1 (3–4 years), GY2 (5–6 years), and GY3 (7–8 years). (**D**) Linear experimental workflow illustrating the complete procedural steps, including field soil sampling, sample pretreatment and fractionation, laboratory quantification and characterization of microplastics (MPs) and heavy metals (HMs), and downstream ecological/health risk modeling.

**Figure 2:**
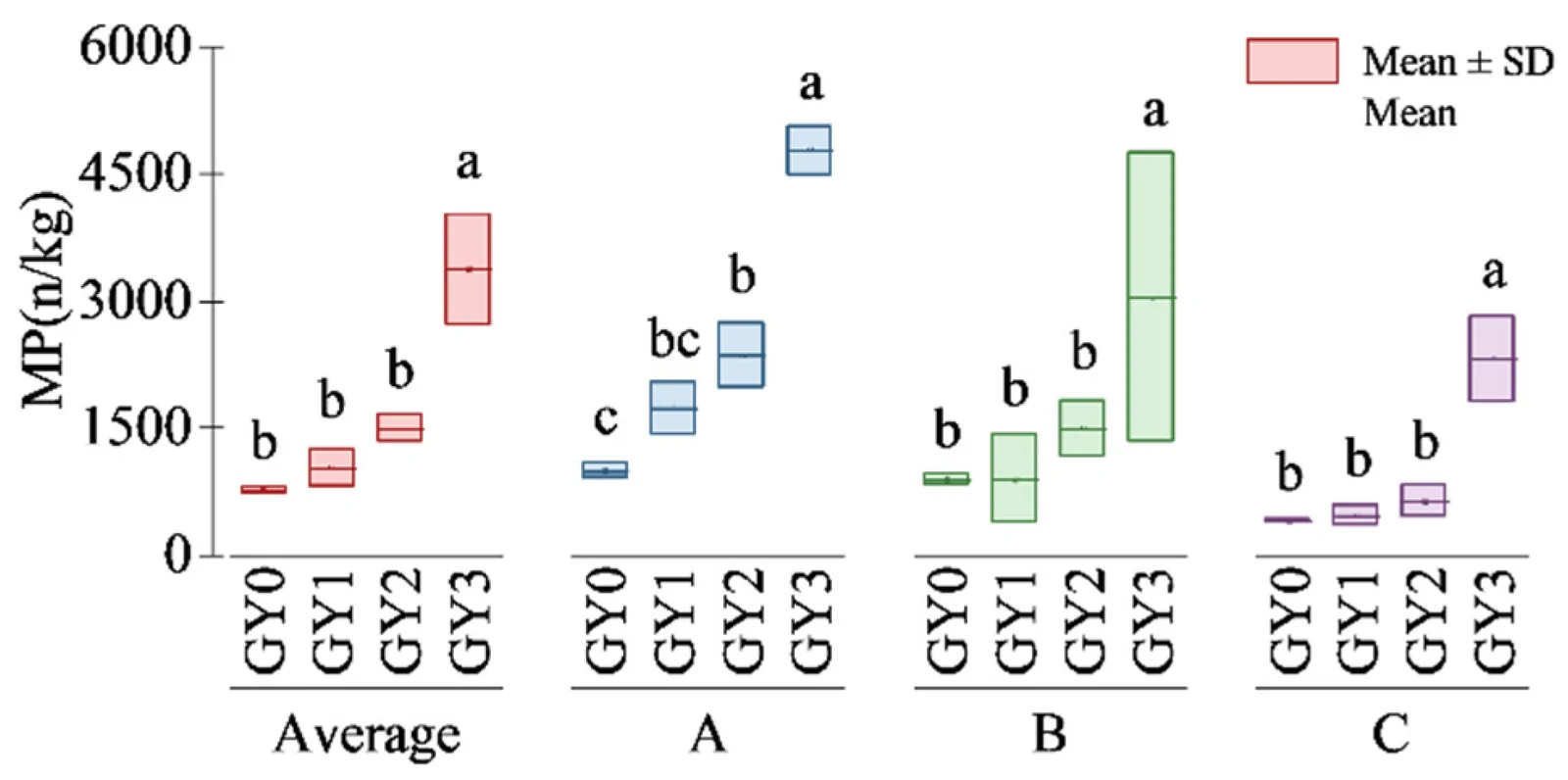
Cumulative changes in MP abudance across soil layers. Boxes indicate the range of mean ± standard deviation (SD), and the dotted line within each box represents the mean, *n* = 3, independent replicate samples per plot per layer. “Average” denotes the overall mean of the three soil layers (A, B, C). Different lowercase letters indicate statistically significant differences among plots of different cultivation years (one-way ANOVA followed by Tukey’s post hoc test, *p* < 0.05).

**Figure 3:**
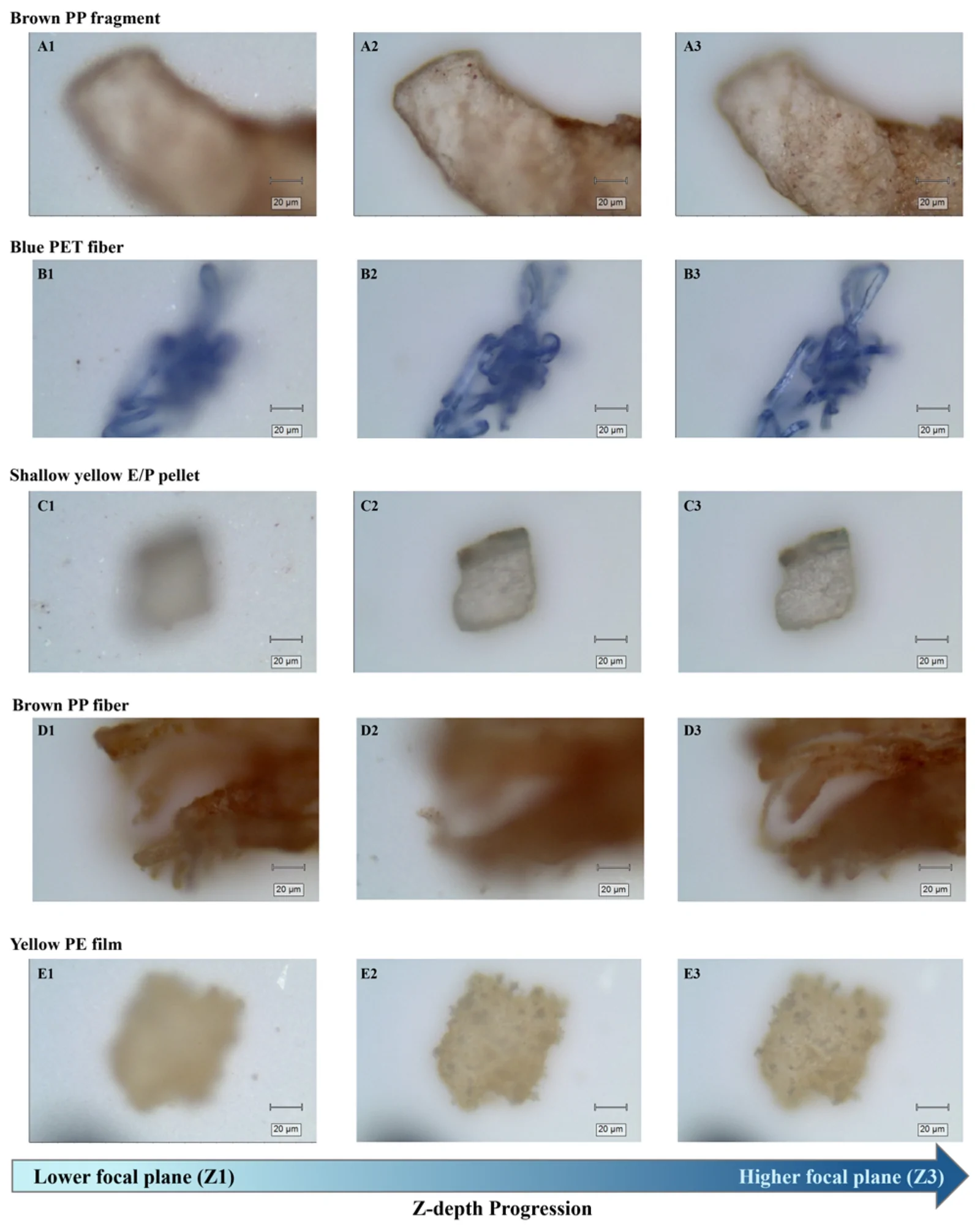
Typical optical morphologies of recovered MP particles under multi-focal plane profiling. Because environmental MPs possess complex three-dimensional geometries and non-planar surface topographies, a single focal plane cannot capture the entire particle in sharp focus without geometric distortion. To ensure accurate spectroscopic identification, multi-plane *Z*-axis focus adjustment was systematically applied during characterization: lower focal planes (**A1**,**B1**,**C1**,**D1**,**E1**) resolved the foundational boundary outlines; intermediate planes (**A2**,**B2**,**C2**,**D2**,**E2**) provided optimal sharpness for targeted Micro-Raman spectral mapping; and upper planes (**A3**,**B3**,**C3**,**D3**,**E3**) captured top-surface weathering features and micro-cracks.

**Figure 4:**
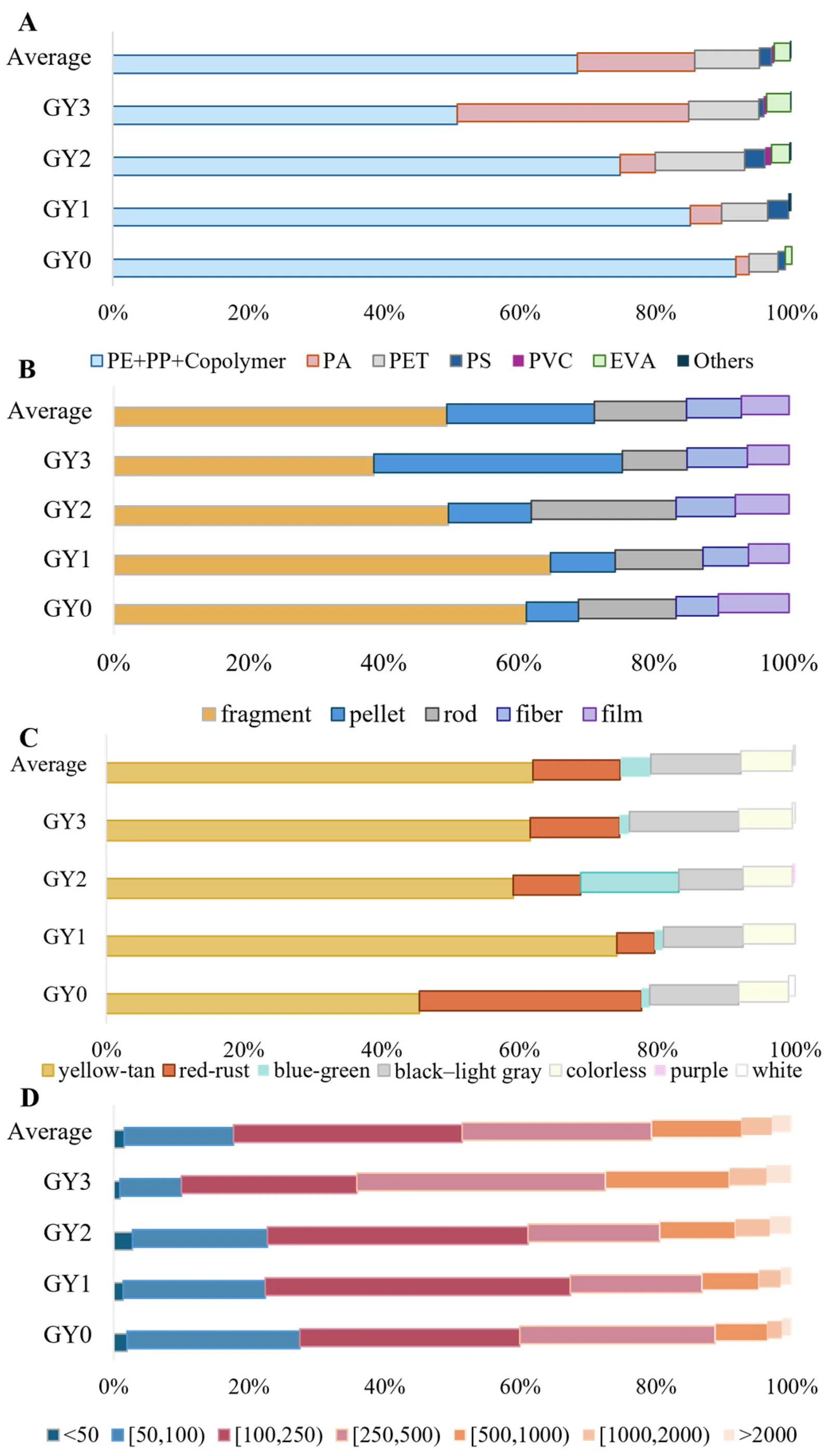
Physicochemical characteristics of microplastics: (**A**) polymer composition, (**B**) particle shape, (**C**) particle-size distribution, and (**D**) color.

**Figure 5:**
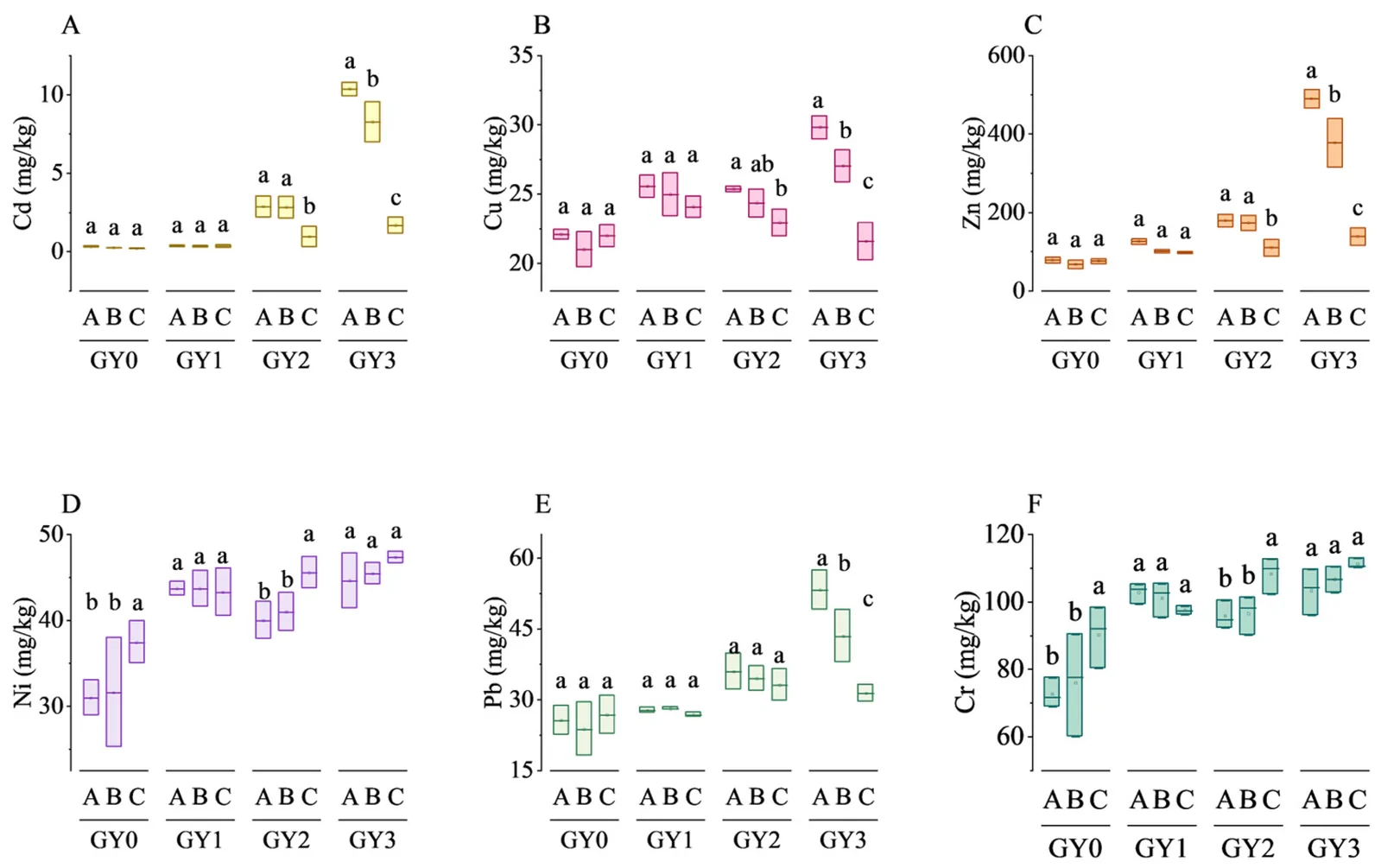
Layer-specific HM concentration for plots in different cultivation years. (**A**): Cd, (**B**): Cu, (**C**): Zn, (**D**): Ni, (**E**): Pb, (**F**): Cr. Boxes indicate the range of mean ± SD, and the dotted line within each box represents the mean. Different lowercase letters indicate statistically significant differences among soil layers (one-way ANOVA followed by Tukey’s post hoc test, *p* < 0.05).

**Figure 6:**
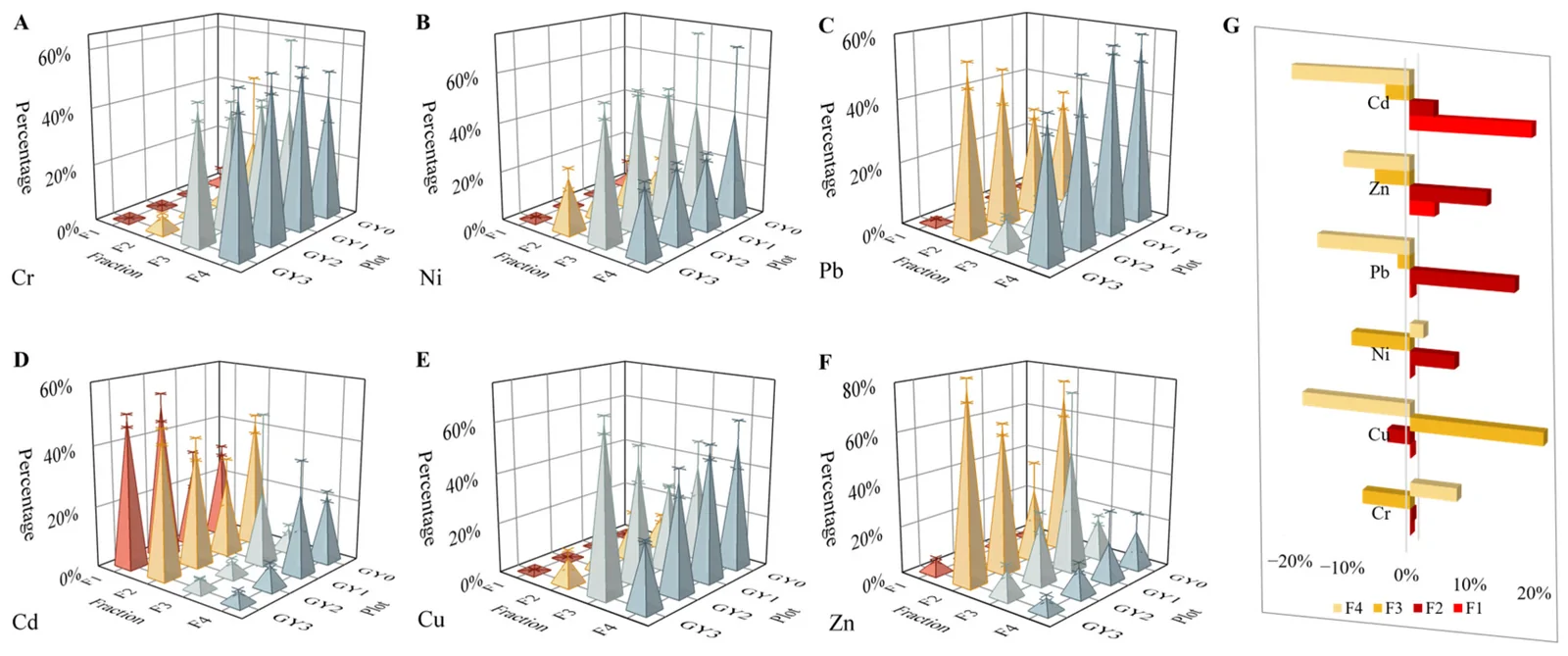
Chemical speciation and temporal shifts in heavy metals (HMs) across microplastics (MP) accumulation gradients. (**A**–**F**) Relative abundances of BCR chemical fractions (F1–F4) for individual heavy metals across cultivation plots. (**G**) Percentage point changes in heavy metal fractions from GY0 to GY3. F1, F2, F3, and F4 represent exchangeable, reducible, oxidizable, and residual fractions, respectively. Data are presented as means ± SDs, *n* = 3, independent replicate samples per plot per layer.

**Figure 7:**
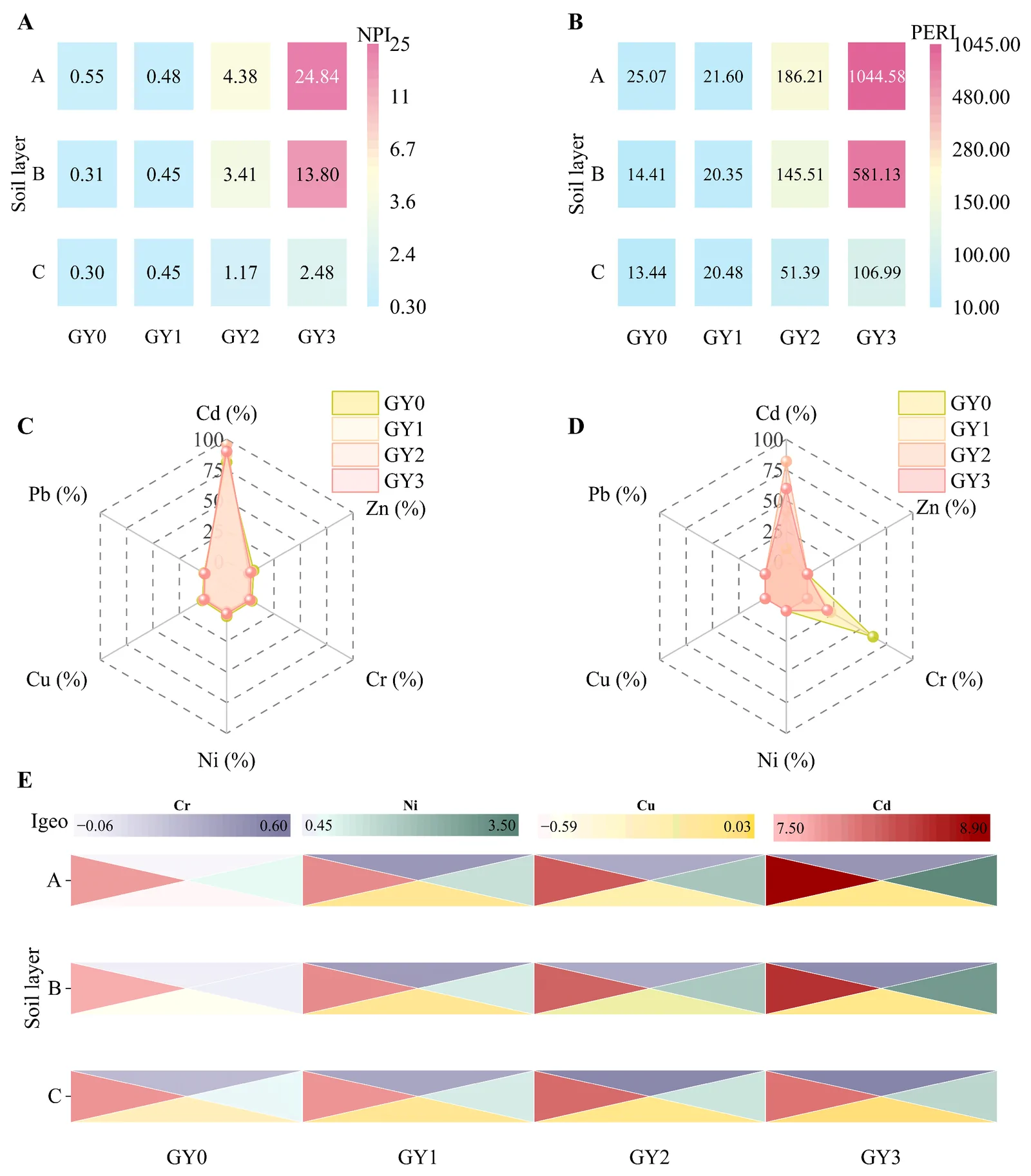
Risk index evaluation and the contribution of individual elements to each index value. Panels (**A**,**B**) are heatmaps showing the comprehensive evaluation results of the NPI and the PERI, respectively. Panels (**C**) display the element contribution analyses for PERI and (**D**) display the element contribution analyses for NPI. The contribution to the NPI was determined by sensitivity analysis, i.e., calculating the percentage change in NPI after removing each element, whereas for the PERI, the contribution was derived directly from the individual potential ecological risk factor (E_r_^i^) of each element. Panel (**E**) illustrates the geo-accumulation index ($I_{geo}$) results, showing only the four elements for which $I_{geo}$ > 0 at any sampling site. Lead (Pb) and zinc (Zn) are not present here as all their $I_{geo}$ values were below zero, indicating clean conditions.

**Figure 8:**
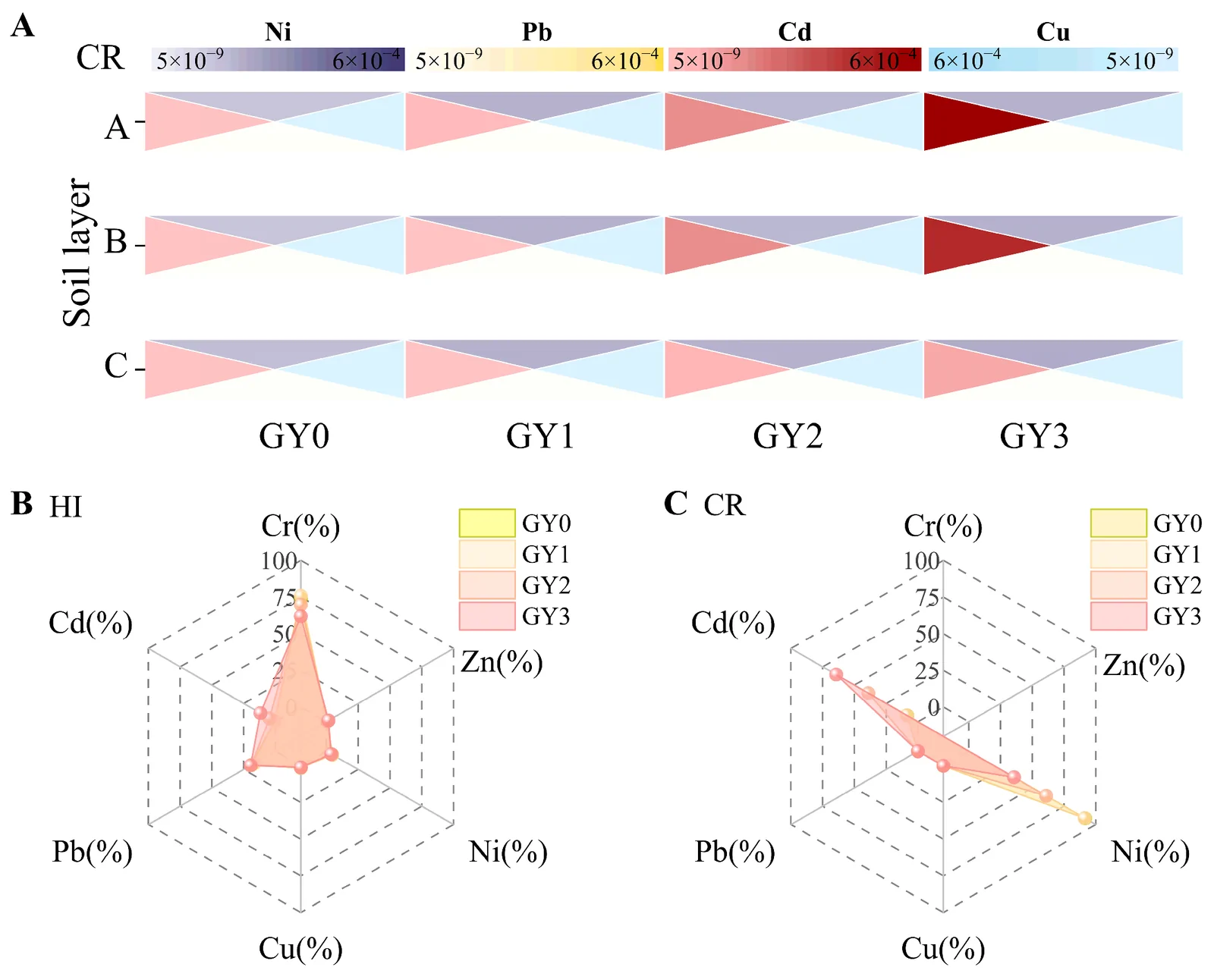
Health risk assessment results. (**A**) Carcinogenic risk (CR) assessment results. (**B**,**C**) show the contributions of individual metal elements to the HI and to the CR, respectively.

**Figure 9:**
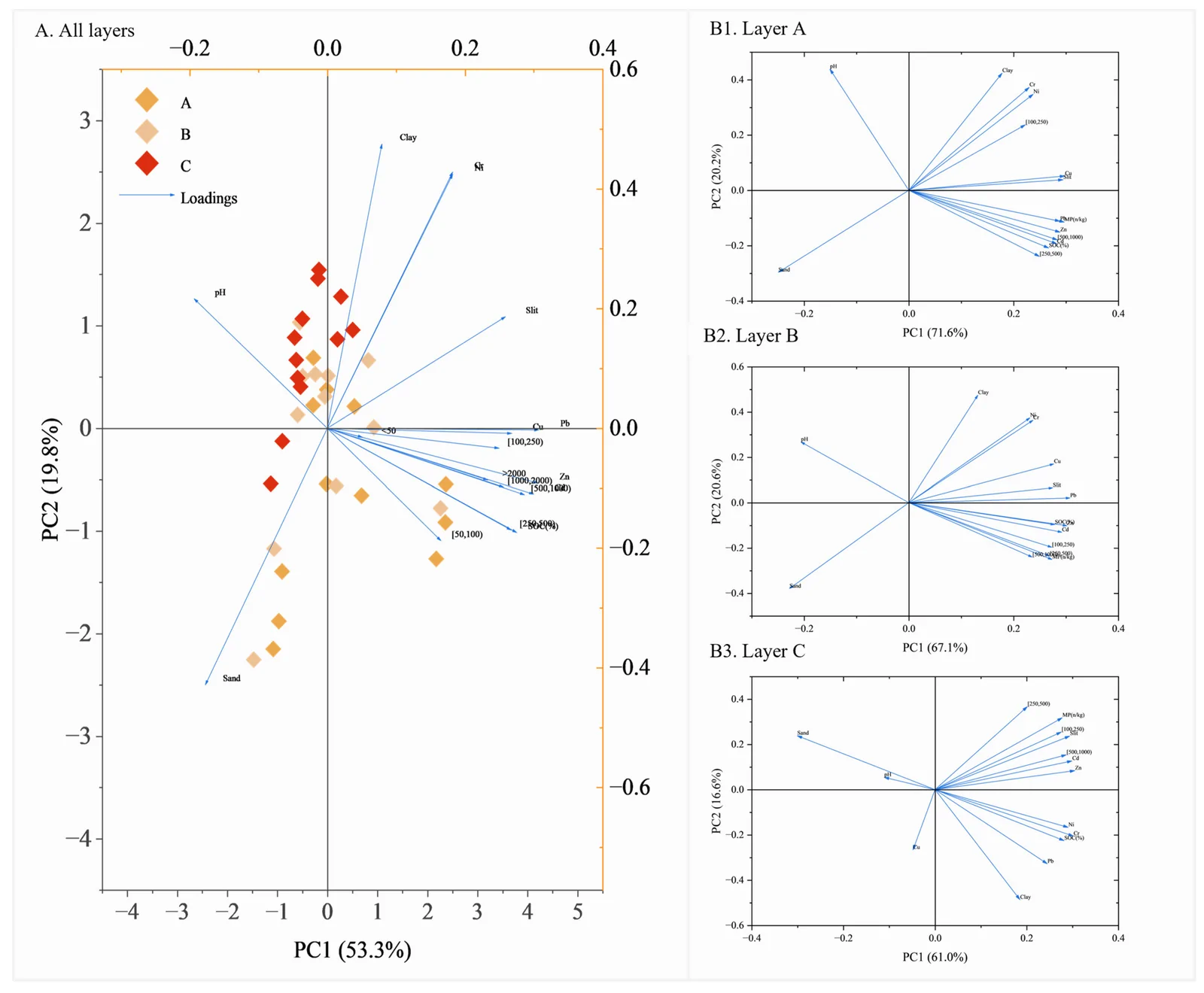
PCA of samples across all depths and individual soil layers. The left panel (**A**) shows the overall PCA for all samples combined, with samples from layers A, B, and C represented by three different colors. The right panel presents (**B1**–**B3**) shows the PCA results for each soil layer separately. Axis labels indicate the percentage of variance explained by each principal component. The length of the arrows represents the strength of interpretation, the arrow direction reflects its spatial loading orientation, and the angle between two arrows represents the correlation between the variables (acute angle: positive correlation; obtuse angle: negative correlation).

**Figure 10:**
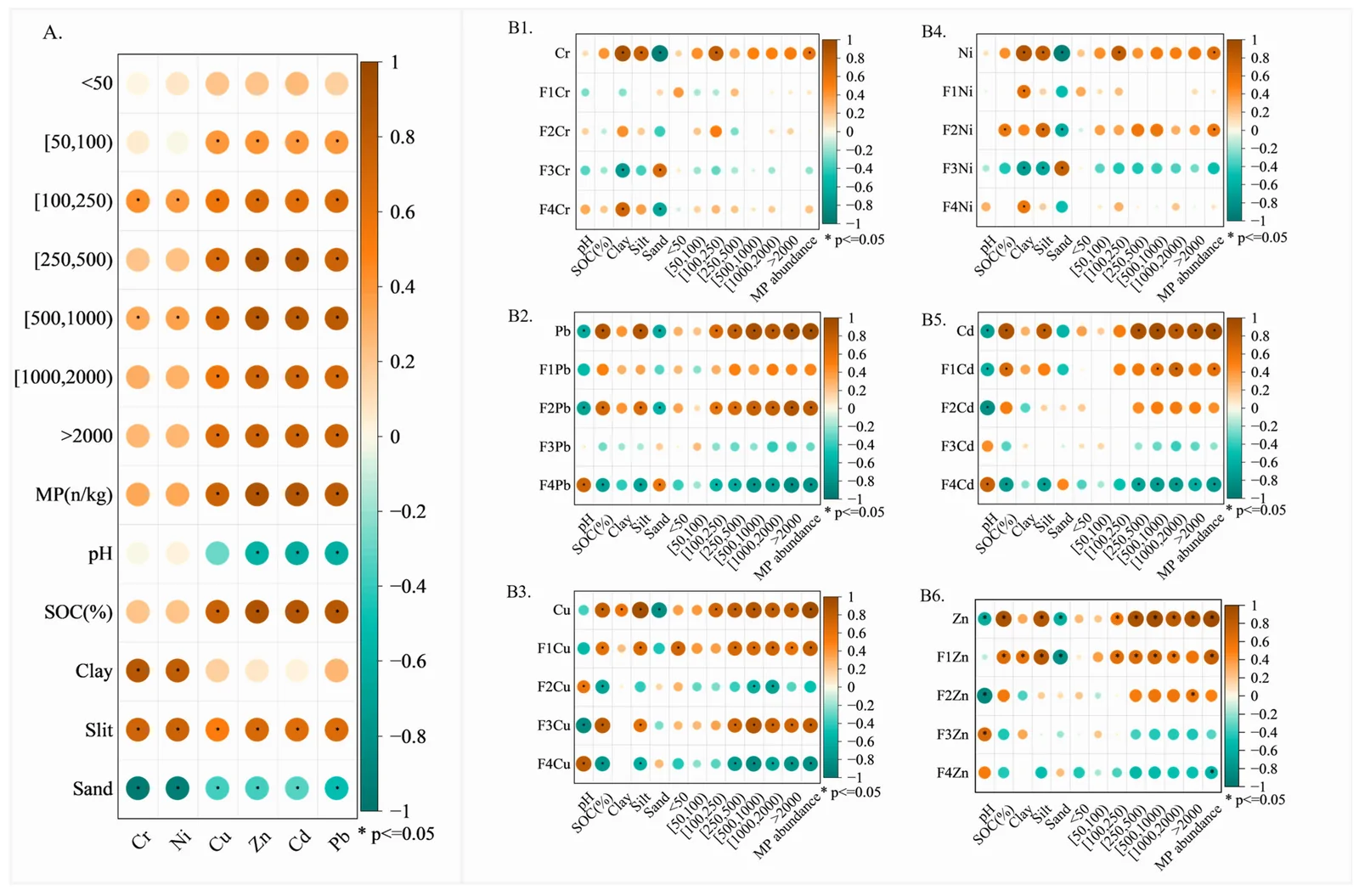
Heatmaps depicting correlations of HMs with soil microplastics and other physicochemical properties. Left panel (**A**) shows the results for samples from all soil layers, where the lower axis indicates the total load of each HM. Because Layer A was identified as the most sensitive layer, the right panel (**B1**–**B6**) was created to analyze the correlations between chemical speciation and other factors in this hotspot; each subfigure represents one metal element ((**B1**): Cr, (**B2**): Ni, (**B3**): Pb, (**B4**): Cd, (**B5**): Cu, (**B6**): Zn). The left axis in the right panel represents the total HM load and the proportions of F1–F4 fractions in Layer A. Positive correlations are shown in brown, and negative correlations in green (*p* set at 0.05).

**Table 1:** HMs statistics and backgroud level.

	Cr	Ni	Cu	Zn	Cd	Pb
Background values	50.50	32.60	31.10	86.50	0.08	30.90
Mean	96.81	41.19	24.22	168.09	2.39	32.48
SD	12.88	5.67	2.61	128.96	3.34	8.75
Min	59.98	25.34	19.67	56.67	0.18	19.80
Max	112.90	47.97	30.59	510.02	10.86	57.85
CV (%)	13.31	13.77	10.77	76.72	139.29	26.94

## Data Availability

The raw data supporting the conclusions of this article will be made available by the authors upon request.

## Supplementary Materials

The following supporting information can be downloaded at https://www.mdpi.com/article/10.3390/toxics14100863/s1, Figure S1: Dynamics of physical and chemical properties; Table S1: Description of chemical analysis for soil physicochemical parameters; Table S2: Grading standards of HM risk index; Table S3: Parameters information used in health risk assessment; Table S4: Reference dose (RfD) and slope factors (SF) values of human exposure to HMs; Table S5: Description of significant correlations between fractions and other factors for Pb, Zn, Cd, Cu [76,77,78].
