# Supplementary material for: Long-Term Microplastic Accumulation Is Associated with Element-Specific Heavy Metal Mobilization in Agricultural Soils: Field Evidence from the Anning Valley, China
Source: Toxics. 2026 Sep 28;14(10):863. doi: 10.3390/toxics14100863 (PMC13709959; doi:10.3390/toxics14100863)
Supplement: Supplementary file 1 [file toxics-14-00863-s001.zip › toxics-4551229-supplementary.pdf]

Supplementary Materials

Long-Term Microplastic Accumulation Is Associated with Element-Specific Heavy Metal Mobilization in Agricultural Soils: Field Evidence from the Anning Valley, China

Yuqing Zhang[abc], Guangli Xiao[d], Junlun Meng[d], Siwei Deng[a], Wenhao Wang[a], Xingguo Hua[a], Xuemei Gou[a], Tianwei He[a], Yi Wu[a,*]

a. Sichuan Academy of Eco-Environmental Sciences, Sichuan Province Engineering Technology Research Center of Emerging Contaminants Treatment and Environmental Health, Chengdu 610041, China

b. College of Ecology and Environment, Chengdu University of Technology, Chengdu 610051, China

c. State Key Laboratory of Geohazard Prevention and Geoenvironment Protection, Chengdu University of Technology, Chengdu 610059, China

d. Sichuan Institute of Energetical and Geological Survey, Chengdu, 610072, China

*. Correspondence: Yi Wu

Table S1 Description of chemical analysis for soil physicochemical parameters

Indicator	Operation
MPs (microplastics)	Briefly, the sample was dispersed in a $ZnCl_2$ solution (density: 1.55 g/mL) before the supernatant containing the buoyant fraction was decanted and filtered through a 20-μm stainless steel sieve. This density flotation step was repeated twice to ensure sufficient recovery. The collected material then underwent Fenton's reagent (30% H_2O_2 + 0.05 M $FeSO_4$) digestion at 40°C to remove natural organic matter. Post-digestion, another round of density flotation and sieving (20 μm) was performed to remove residual impurities. The final extract was vacuum filtered onto a mixed cellulose ester (MCE) membrane filter (pore size < 1 μm) for collection. MP characterization was performed via coupled microscopy-Raman spectroscopy. Suspected particles were analyzed by Raman spectroscopy (785 nm laser excitation, 50× objective) and identified by a match quality index (HQI) [41].
pH	measured potentiometrically using a pH electrode (PHS-3C, Shanghai Yidian Scientific Instrument Co., China) in a soil-to-deionized water suspension (1:2.5, w/w).
Soil moisture content (SWC)	determined gravimetrically as a subsample in an aluminum box that was dried at 105°C to constant weight.
Soil organic carbon (SOC) content	analyzed by the Walkley-Back wet oxidation method [76].
Soil texture (mechanical composition)	including the percentages of clay, silt, and sand, determined by pipette method and densitometry [77, 78].

Table S2 Grading standards of HM risk index [46]

Pi and *NPI*	Classification	E_r^i	Classification	*PERI*	Classification	I_{geo}	Classification
<1	clean	<40	low	<150	low	<0	clean
(1,2]	low	(40,80]	moderate	(150,300]	moderate	(0,1]	slight
(2,3]	moderate	(80,160]	considerable	(300,600]	high	(1,2]	moderate
>3	high	(160,320]	high	>600	very high	(2,3]	alert
		>320	very high			>3	heavy

Table S3 Parameters information used in health risk assessment

Parameter	Description	Unit	Children	References
IngR	Ingestion rate of soil	mg/day	200	
InhR	Inhalation rate of soil	m^3/day	7.5	
EF	Exposure frequency	day/year	350	
ED	Exposure duration	year	6	[54]
BW	Body weight	kg	19.2	
AT	Average time	day	365*ED	
PEF	Particle emission factor	m^3/kg	1.36E+09	

SA	Skin area available for soil contact	cm^2	2373
AF	Soil-to-skin adherence factor	$mg/cm^2/day$	0.2
ABS	Absorption factor	-	0.001

Table S4 Reference dose (RfD) and slope factors (SF) values of human exposure to HMs

	RfD(mg/(kg · d)			SF((kg · d)/mg)			Reference
	ingestion	inhalation	dermal contact	ingestion	inhalation	dermal contact	
Cr	3.00E-03	2.86E-05	7.50E-05	-	-	-	[51-53]
Zn	3.00E-01	3.00E-01	6.00E-02	-	-	-	
Ni	2.00E-02	9.00E-05	8.00E-04	5.00E-01	4.20E+01	1.25E-02	
Cu	4.00E-02	4.02E-02	1.20E-02	-	8.40E-01	-	
Pb	3.50E-03	3.52E-03	5.25E-04	-	6.30E+00	-	
Cd	1.00E-03	2.86E-06	2.50E-05	6.10E+00	6.30E+00	6.10E+00	

Note: SF (slope factor) characterizes the carcinogenic potency of a heavy metal via different exposure routes, and RfD (reference dose) is the maximum daily exposure level for a given route that is likely to be without an appreciable risk of adverse health effects over a lifetime. Here the oral carcinogenic slope factors for Ni and Cd were adopted as a screening-level, highly conservative estimate based on previous literature [51, 52].

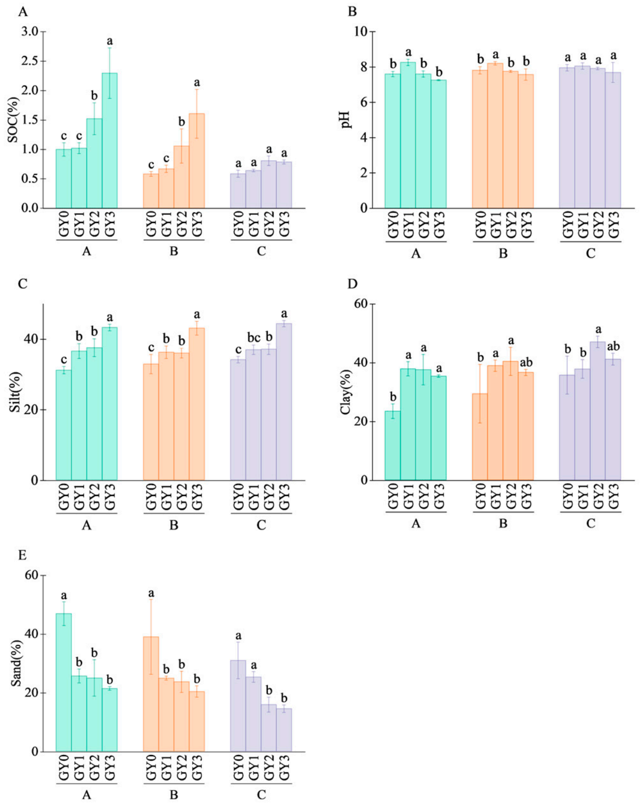

Figure S1 Dynamics of physical and chemical properties. A:SOC,B:pH,C:Slit percentage(%),D:Clay percentage (%) and E:sand percentage (%). Data are presented as means ± SDs, $n = 3$, independent replicate samples per plot per layer. Different lowercase letters indicate statistically significant differences among plots of different cultivation years (one-way ANOVA followed by Tukey's post-hoc test, $p < 0.05$)

Table S5 Description of significant correlations between fractions and other factors for Pb, Zn, Cd, Cu

Heavy metals	Correlation between HM fractions and other factors
Pb	The dominant mobile fraction (F2) showed significant positive correlations ($P < 0.05$) with MP abundance ($r = 0.81$), SOC ($r = 0.72$), and silt content ($r = 0.68$), and significant negative correlations ($P < 0.05$) with pH ($r = -0.70$) and sand content ($r = -0.59$).
Zn	Its mobility was influenced by multiple factors. The reducible fraction (F2) correlated positively with SOC ($r = 0.55$) and MPs ($r = 0.49$), and negatively with pH ($r = -0.87$). The acid-soluble fraction (F1) correlated positively with silt ($r = 0.85$) and negatively with sand ($r = -0.80$) (all $P< 0.05$).
Cd	The proportions of its mobile fractions (F1+F2) correlated positively with both MP abundance (F1: $r = 0.63$; F2: $r = 0.42$) and SOC (F1: $r = 0.69$; F2: $r = 0.53$). Most fractions (except F3) showed significant negative correlations with pH, strongest for F2 ($r = -0.81$). Correlations with soil texture were generally weak, with only a significant negative correlation ($P < 0.05$) between the residual fraction (F4) and silt content ($r = -0.70$)
Cu	The shift from residual (F4) to oxidizable (F3) fraction was significant ($P < 0.05$). The F3 fraction correlated positively with SOC ($r = 0.82$) and silt content ($r = 0.64$), and negatively with pH ($r = -0.85$).
